# After we transgress God’s values: relational spirituality as a paradigm for the Christian experience of reconciliation with God

**DOI:** 10.3389/fpsyg.2025.1587704

**Published:** 2025-05-09

**Authors:** Kaye V. Cook, Richard G. Cowden

**Affiliations:** ^1^Gordon College, Wenham, MA, United States; ^2^Human Flourishing Program, Harvard University, Cambridge, MA, United States; ^3^Department of Epidemiology, T.H. Chan School of Public Health, Boston, MA, United States

**Keywords:** divine forgiveness, relational spirituality, repentance, absolution, communion with God, personal sin

## Abstract

In the Christian spiritual journey, the desire for communion with God is sometimes challenged by personal sin—instances in which the believer transgresses God’s standards or values for how they ought to live. Thus, the need for divine forgiveness is an important concept in Christianity. To date, relatively little research has explored Christian *experiences* of forgiveness from God for personal sin, the factors that shape those experiences, and the effects of experiencing forgiveness from God on different aspects of their life. To support the development of research in this area, this paper proposes a relational spirituality model of personal sin for conceptualizing how a network of interrelated cognitive-affective appraisals involving the self, the personal sin, God, and other people can shape a Christian’s experience of, response to, and resolution of personal sin. We introduce the core elements of the model and explore how the interrelations among these elements might influence a Christian’s relational experience with God following recognition of a personal sin. We discuss the unique contributions of the model to the psychological literature on divine forgiveness and highlight implications of the model for advancing research on Christian experiences of forgiveness from God.

## Introduction

Most people around the world identify with a theistic religious tradition in which the object of faith is a supreme being or higher power that forgives (Pew Research Center, 2012, 2015). Christianity, comprising roughly one-third of the global population, is the largest of these religious traditions and has a rich theology about forgiveness from God (Cowden et al., 2023; Rutledge, 2022). For example, Christians are encouraged to seek absolution from God for their personal sin, a process that is fundamental to the Christian religion and emphasized throughout the Bible, the source of Christian teachings, beliefs, and practices. One illustration of this is found in 1 John 1:9, which says: “If we confess our sins, he is faithful and just and will forgive us our sins and purify us from all unrighteousness” [New International Version (NIV), 2011].

Despite the central importance of divine forgiveness to the Christian tradition and evidence suggesting that divine forgiveness has potential implications for well-being across spiritual, mental, social, and physical dimensions of life (Fincham and May, 2022; Long et al., 2020), relatively little empirical research has explored Christian *experiences* of forgiveness from God, the factors that shape those experiences, and the effects of experiencing forgiveness from God on different aspects of a person’s life. To support empirical research in this area, this conceptual paper draws on insights from relational spirituality to outline a theoretical model that captures how Christians might appraise, respond to, and resolve instances in which they have violated God’s standards or values.

### Communion with God: a central aim of the Christian life

At the heart of the Christian life lies a desire for communion with God, a mutual, intimate, dynamic relationship with God that is initiated by God and reciprocated by Christians, resulting in biopsychosocial-spiritual benefits (Knabb and Wang, 2021; Koenig et al., 2024). This desire for communion with God may be expressed through prayer, attending religious services, developing deep relationships with others who share the same desire, and/or studying the Bible or other religious materials.

Communion with God has both doctrinal and experiential dimensions. The doctrinal dimension pertains to ways that believers view and understand God, while the experiential dimension focuses on how Christians personally experience God (Davis et al., 2021; Knabb and Wang, 2021). For instance, the belief that God forgives is part of the doctrinal dimension, whereas the feeling of being forgiven can be situated within the experiential dimension.

Doctrinally, Christians believe that divine forgiveness is received through Jesus Christ, as described in the Bible and other teachings in the Christian church. One illustration of this can be found in the Nicene Creed, a core statement of orthodox belief within the Christian tradition, which states: “we acknowledge one baptism for the forgiveness of sins.”^1^ Despite the doctrinal significance of divine forgiveness, believers can sometimes struggle to accept or experience forgiveness from God (Cowden et al., in press). One way in which dissonance between the doctrinal and experiential dimensions of forgiveness from God might arise is when an individual becomes consciously aware of personal sin.

Personal sin^2^ involves ‘turning away’ from God through thoughts or behaviors that transgress God’s standards or values for how people ought to live (McCall, 2019). From a theological perspective, then, personal sin is inherently relational. There are different ways to classify personal sin. One common schema distinguishes between sins of commission (i.e., willfully thinking, doing, or saying something that is contrary to God’s values) and sins of omission (i.e., when a person fails to do something that God commands or expects). Personal sins may be committed alone (e.g., acting vengefully toward another person) or with another person (e.g., when two people conspire to falsely accuse another person). Some Christian traditions (e.g., Roman Catholicism) differentiate between mortal (or grave) personal sins that separate one from God and venial (or lesser) personal sins that damage but do not sever one’s relationship with God.

Christians show between- and within-person differences in how they encounter their personal sin. For instance, one person may perceive themselves as having grievously transgressed God’s values, whereas another who commits a similar personal sin might minimize its severity. Such between-person differences may themselves vary denominationally due to differences in theological beliefs, scriptural interpretation, and emphasis on different aspects of doctrine. For example, a person who is part of a Christian tradition that practices sacramental confession (i.e., confession of personal sin to a priest), such as Roman Catholicism, might be more likely to confess their personal sin to others in the religious community than someone from a denomination (e.g., Baptist) that encourages direct confession to God. It is also possible that a person who considers themselves to have committed a minor personal sin at one point in their life might be deeply grieved over that same personal sin as they mature spiritually, highlighting potential within-person variation over the life course.

When someone becomes consciously aware of their personal sin, they may respond in multiple ways (Cowden et al., in press). For example, a person may attempt to justify their actions, thereby undermining their efforts toward spiritual growth. Alternatively, they may acknowledge that their actions have violated God’s standards or values, potentially leading to experiences of guilt and shame, which might prompt a renewal of their motivation to pursue spiritual disciplines (e.g., prayer). This dissonance might be conceptualized as a form of religious/spiritual struggle (i.e., tension, strain, or conflict around sacred matters), which can bring about a perceived change in their sense of communion with God.

A Christian may attempt to resolve this state of relational disharmony with God by employing one or more coping strategies (Worthington et al., 2019). For instance, a Christian may cope with their personal sin by moving closer to God (e.g., confessing the personal sin to God) or by withdrawing further from God (e.g., deciding to no longer read the Bible). Movements toward God signal repentance, reflecting the notion of turning back toward God after having turned away through personal sin (Heimann, 2022). Engaging in repentance with God is a multidimensional process involving cognitive, affective, or behavioral components that interact dynamically to shape experiences. From a broadly ecumenical perspective, some of the key aspects of engaging in repentance with God include admitting personal wrongdoing to God, experiencing remorse for violating God’s values, and renewing a commitment to live in accordance with God’s values (Luban, 2023; Worthington et al., 2019).

The process of restoring relational harmony with God, often begun through repentance, also involves a parallel process in which a Christian perceives God moving toward them as they experience divine absolution for personal sin. The multidimensional process of experiencing absolution from God for personal sin involves change from a negative state of unexperienced forgiveness from God to a positive state of experienced forgiveness from God for that sin. Central features of this process might include feeling cleansed by God from the guilt experienced because of the personal sin, coming to experience peace with God, feeling as though God has freed them from its burden, and experiencing a sense of assurance that God has completely forgiven them (Luke 15:11–32; Worthington et al., 2019). The dual processes of engaging in repentance with God and experiencing absolution from God are thought to bring about improved relational harmony with God, which might be referred to as experiencing reconciliation with God (Cowden et al., in press). This experience likely paves the way for deeper communion with God and enhanced well-being.

### The relational spirituality model of personal sin

Relational spirituality is concerned with ways of relating to the sacred. Two of its underlying assumptions are that the individual believes in God and can have a relationship with God (Sandage and Shults, 2007; Shults and Sandage, 2006). Relationships can take many forms—one can revere God, talk with God and expect a response, or seek God’s company. Christianity is not unique in its emphasis on having a relationship with God; other theistic religions (e.g., Judaism, Islam) carry a similar emphasis and adherents may also characterize God as benevolent (Silverman et al., 2016; Van Cappellen et al., 2024).

The relational spirituality system outlines a network of interconnected factors that influence a Christian’s sense of communion with God (Shults and Sandage, 2006). Relational spirituality emphasizes the role of relationships in shaping a person’s spirituality, which might be defined as “ways of relating to the Sacred” (Worthington and Sandage, 2016, p. 38). Although the landscape of this complex and dynamic system can be carved up in different ways, the relational spirituality system includes numerous elements across intrapersonal (e.g., assumptions about reality, Christian identity), interpersonal (e.g., assumptions about the nature of people, relationships with others), and transpersonal (e.g., beliefs about God’s character, past experiences attributed to God) dimensions of a person’s life. These elements play a crucial role in forming, maintaining, and nurturing a Christian’s spiritual connection to God; however, there may be times when certain elements within the system thwart or disrupt a person’s relational harmony with God.

The system’s default state is relative homeostasis, meaning a somewhat stable internal condition despite external changes. In relational spirituality, this stability may reflect a sense of spiritual dwelling (e.g., experiencing security in God’s presence) that fosters communion with God (Wuthnow, 1998; Worthington and Sandage, 2016). Awareness of personal sin can disrupt this relative stability. Disturbance introduces spiritual disharmony, unsettling the Christian’s experience of communion with God. To resolve this disharmony, individuals might engage in spiritual seeking, actively searching for new spiritual meaning and a sense of peace in their efforts to restore harmony within the relational spirituality system (Cook et al., 2014; Wuthnow, 1998). This process may ultimately lead to experiencing reconciliation with God (Cowden et al., in press). Although the notion of experiencing reconciliation with God following recognition of personal sin rightly makes the individual’s relationship with God the central point of emphasis, relational spirituality suggests that a person’s experience of reconciliation with God necessarily depends on a broad network of interrelationships involving the self, God, the personal sin, and other people. Drawing on prior literature concerning relational spirituality and interpersonal forgiveness (see Davis et al., 2008; Shults and Sandage, 2006; Worthington and Sandage, 2016), we introduce a *relational spirituality model of personal sin* that provides a psychological framework for conceptualizing how a Christian’s relationship with God following recognition of a personal sin unfolds within a broader network of interrelated elements.

**Figure 1:**
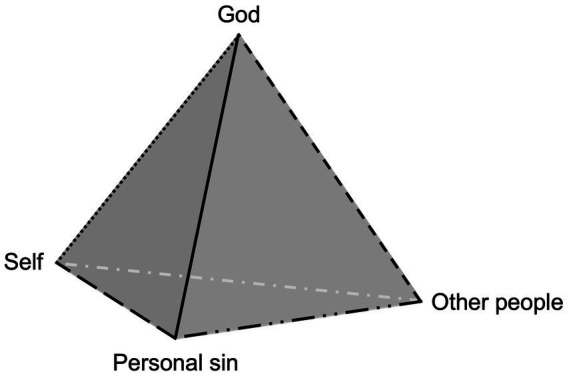
The model depicts a psychological framework for conceptualizing how a Christian’s relationship with God unfolds following recognition of personal sin. The components include the transgressor’s appraisals of (1) four elements that are of primary importance in the aftermath of recognizing personal sin (i.e., the self, the personal sin, God, other people) and (2) the relationships between these elements, which together contribute to shaping their experience of reconciliation with God. The corners (or nodes) of the prism denote the transgressor’s appraisals of the elements; the lines (or edges) denote the transgressor’s appraisals of the relationships among the elements.

We illustrate this model using a triangular pyramid (see Figure 1) involving the self^3^, the personal sin, God, and other people who are directly or indirectly connected to the personal sin^4^. The vertices (or nodes) correspond to the person’s cognitive-affective appraisals involving these four elements. The lines (or edges) linking each node correspond to the person’s cognitive-affective appraisals of the relationships between (1) the self and God, (2) the self and personal sin, (3) the self and other people, (4) God and the personal sin, (5) God and other people, (6) the personal sin and other people. Importantly, the model considers not only one’s perceived relationship with God but also how a broader set of relational dynamics—with the self, the personal sin, and other people—might alter a Christian’s resolution of personal sin. Consistent with prior work on interpersonal forgiveness (e.g., Worthington and Sandage, 2016), the concept of appraisals encompasses the key ingredients—such as cognitive evaluations and emotional responses—that motivate and calibrate the person’s response to the disturbance in their sense of communion with God caused by recognition of personal sin and are especially relevant to experiencing reconciliation with God. Appraisals may be interconnected node-to-node (e.g., connections between appraisals of the self and appraisals of others), node-to-edge (e.g., connections between appraisals of the self and appraisals of one’s relationship with God), or edge-to-edge (e.g., connections between appraisals of one’s relationship to the personal sin and one’s relationship with God).

We unpack the relational spirituality model of personal sin under the assumption that the system of interrelationships between cognitive-affective appraisals involving God, the self, the personal sin, and other people is disturbed from its state of relative homeostasis by personal sin entering a Christian’s conscious awareness. Personal sin may enter one’s awareness in multiple ways: one may be convicted by the Holy Spirit (John 16:8), read scripture and feel guilty (Romans 10:17), experience personal hardship (Psalm 119:67), or be chastised by another person (Acts 8:20). Members of one’s religious community may help a person to acknowledge their personal sin, perhaps by reminding them of their responsibility to the community or by helping the wrongdoer feel accepted within the community despite their personal sin. Some Christian denominations (e.g., Calvinism) teach that God’s grace is so powerful it cannot be resisted (i.e., irresistible grace); when God offers his saving grace, it is thought to trigger a transformation of a person’s heart that leads them to repent. Although disruption to homeostasis in a relational spirituality system may occur through a variety of mechanisms, the relational spirituality model of personal sin is principally concerned with how recognizing personal sin disrupts the relational spirituality system, the subsequent changes within the system, and the processes involved in restoring relative homeostasis. We focus primarily on appraisals that might increase communion with God, but we also recognize that a person’s appraisals may maintain or even exacerbate tension within the relational spirituality system that might not result in renewed communion with God.

#### Nodes and edges

A person who realizes they have transgressed against God’s standards or values appraises the elements (or nodes) in their relational spirituality context that shape their experience of reconciliation with God. Our proposed model focuses on appraisals related to four interconnected elements, one of which is the self. For example, prior research suggests that self-appraisals play a role in how individuals respond to personal wrongdoing. People with low self-esteem are more prone to shame, which can hinder their ability to restore self-worth (Cowden et al., 2020; Strelan, 2007). This may, in turn, create a barrier to experiencing absolution from God (Abernethy et al., 2022).

Another central element involves appraisals pertaining to God. For instance, a Christian may perceive God as benevolent and quick to forgive or as an angry, wrathful God who does not readily forgive. These appraisals may shape a person’s general perspective on the conditionality of forgiveness from God, which could affect whether they seek forgiveness from God following recognition of personal sin (Fincham, 2024; Fincham and Maranges, 2025).

Christians appraise the nature of their personal sin, which represents the third node in our proposed model. Some individuals are more inclined to acknowledge personal sins than others (Ludwig et al., 2025). Appraisals involve various dimensions, including the perceived severity of the personal sin, which has been shown to predict the likelihood of seeking divine forgiveness (Fincham and May, 2023). People may respond to personal wrongdoing in different ways—by denying they have done wrong, justifying their actions as morally acceptable, excusing their actions, or acknowledging their wrongdoing (Schönbach, 1990). Additionally, appraisals of the nature of one’s sin, such as identifying one’s personal sin as a religious/spiritual struggle, can shape how individuals perceive and address the consequences of their transgressions (Exline et al., 2014).

Transgressors also make appraisals of others who are connected to the personal sin in some way, which is the fourth element in our proposed model. When transgressors realize their personal sin has hurt someone, empathy-related appraisals (e.g., perceived distress experienced by the victim) may lead them to engage in amends-making behavior, seek forgiveness, and pursue reconciliation (Worthington and Wade, 2020). Even when another person is not directly involved in or affected by the personal sin, transgressors often consider the impact of their actions on others. Social relationships can influence transgressors’ decision-making and coping responses following personal sin, particularly in relation to moral judgments and feelings of guilt (Luck and Luck-Sikorski, 2022; Pilcher and Smith, 2024).

Other individuals who are relevant to the transgression can be present in multiple ways (e.g., as collaborators in the personal sin, direct victims of it, or members of one’s religious community who might be involved in addressing it or its consequences). The presence and actions of other individuals can either facilitate or hinder the process of experiencing reconciliation with God through their influence on the transgressor’s appraisals. For instance, when multiple people are co-conspirators in a personal sin, responsibility may become diffused, reducing the likelihood that people will take appropriate personal responsibility for the transgression (Tosuntaş, 2020). Despite the social dimension of personal sin, research on divine forgiveness often overlooks the role of other people in how a transgressor approaches and responds to personal sin. For example, in their analysis of the divine forgiveness process, Fincham and May (2023) acknowledge cases where a transgression involves a victim but otherwise do not consider the role of other people in the process^5^.

In the relational spirituality model of personal sin, appraisals involving the connections (or edges) between elements can also play a crucial role in determining the stability of the relational spirituality system after recognition of personal sin (Fincham and Maranges, 2024; Granqvist et al., 2010; Kent et al., 2018). For instance, a Christian who has a secure attachment with God—a deep, trusting, warm relationship with God in whom they feel safe and cared for—is more likely to appraise other people positively. As a result, they are more likely to form secure attachments to others, perceiving them as safe and trustworthy. They are also more inclined to seek and experience divine forgiveness, whereas those with an avoidant attachment style are less likely to do so.

Personal sin may trigger multiple appraisals involving nodes and edges. For example, Baumeister et al. (1995) found that offenders tend to experience more intense and persistent guilt when they transgress against close friends (a node and an edge appraisal). Along similar lines, Tangney et al. (2007) documented that recognition of personal wrongdoing can trigger cognitive-affective appraisals of the nature of the self and of one’s moral and social responsibility to others (a node and an edge appraisal). Node-to-edge interrelations occur, in some cases during a cost–benefit analysis. Upon recognition of personal sin, a Christian may evaluate their actions as morally unacceptable (an appraisal of the personal sin) and as impacting their relationship with God (an edge appraisal), but at the same time as requiring too much self-control (a self-appraisal) and as potentially harmful to relationships with friends (an edge appraisal) if they discontinue the behavior. Such internal appraisals may lead them to justify, rationalize, or downplay the personal sin or to decide that the benefit of confronting and appropriately addressing the personal sin is worth the cost.

Research suggests that individuals often use their own beliefs (a self-appraisal) as a reference point for understanding the beliefs of others (an appraisal of others). For instance, Epley et al. (2009) found that a person’s egocentric beliefs are associated with their estimates of the beliefs of God and other people. A similar conceptual approach appears in research on the relational spirituality model. To illustrate, Davis et al. (2008) showed that victims of interpersonal transgressions assess the transgressor’s relationship with God by measuring its perceived similarity to the victim’s spirituality. Acknowledging these kinds of connections in the context of personal sin can enhance our understanding of how individuals process their transgressions against God’s standards or values, evaluate systemic elements in the experience, and move toward experiencing reconciliation with God.

Some personal sins may be more disruptive of the relational spirituality system than others. Moreover, certain nodes or edges may play a stronger role in either stabilizing or destabilizing the system. Understanding how individuals assess these relationships—and the relative importance of different nodes and edges—can offer insights that help refine interventions aimed at adaptively strengthening the homeostasis of the relational spirituality system after recognizing personal sin.

#### A biblical example

The biblical story of King David and his interaction with the prophet Nathan gives an example of how the relational spirituality model of personal sin might be applied. In 2 Samuel 12:1–13, Nathan uses a parable of a rich man and poor man to show David that his actions—engaging in sexual relations with Bathsheba and orchestrating the death of her husband—were personal sins. David expresses his remorse by confessing, “I have sinned against the Lord” (2 Samuel 12:13, NIV).^6^ In this case, Nathan’s parable and subsequent interaction with David appear to play a key role in shaping the appraisals of other nodes presented in Figure 1. It is only after David listens to Nathan (the ‘other’ node) that David becomes aware of his personal sin (the ‘personal sin’ node). This awareness then triggers feelings of guilt (the ‘self’ node) and a desire to confess to God, after recognizing that he has violated God’s values (the ‘God’ node). This illustrates how appraisals of one node in the proposed model can cascade through the system, eliciting changes in other nodes, some of which occur temporally sooner than others.

After David’s confession, Nathan pronounces that God has absolved David of his personal sin (2 Samuel 12:13), which likely effected changes to appraisals involving several nodes of the system (e.g., his understanding of God’s character, his perception of himself). Although changing appraisals of any node in the system can ripple through the relational spirituality system, recognition of personal sin may have particularly important implications for the system because it plays such a key role in disrupting the central objective of the Christian’s life—experiencing communion with God.

So far in this example, we have focused primarily on the impact of activating nodes in the relational spirituality system, but cognitive-affective appraisals involving edges in the model can also impact connections among elements in the system. The way the story is told in 2 Samuel 12:1–13 suggests that Nathan’s confrontation of David precipitates a change in David’s appraisal of his personal sin that alters his appraisal of how God perceives his actions (the edge linking the ‘God’ and ‘personal sin’ nodes). David’s appraisal of Nathan’s relationship with him (the edge linking the ‘self’ and ‘other person’ nodes) and with God (the edge linking the ‘God’ and ‘other person’ nodes) presumably plays into his readiness to respond positively to Nathan’s confrontation.

#### Summary

As we have tried to illustrate, instances in which a Christian becomes consciously aware of a personal sin may introduce tension in their relational spirituality system through a disturbance to the network of interrelated cognitive-affective appraisals pertaining to the nodes (i.e., the self, God, the personal sin, and other people with direct or indirect ties to the personal sin) and the relationships among these nodes. Although there are multiple paths through which relative homeostasis may be restored in the system following recognition of personal sin, Cowden et al. (in press) suggest the dual processes of engaging in repentance with God and experiencing absolution from God—each of which is shaped by the relational spirituality dynamics outlined in our proposed model—may be necessary for improving homeostasis within a Christian’s relational spirituality system in service of a renewed or strengthened sense of communion with God.

## Discussion

The Christian life centers on a desire for communion with God (Knabb and Wang, 2021). When an instance of personal sin enters a Christian’s conscious awareness, it may trigger dissonance between the doctrinal and experiential dimensions of their spiritual life, potentially leading to disharmony in their relationship with God (Cowden et al., in press). Although there are numerous biblical accounts and anecdotes of Christians experiencing reconciliation with God in the aftermath of personal sin (e.g., Luke 23:39–43, John 21:15–19), psychologically grounded empirical evidence documenting this process is limited and needs further attention.

To support the development of research in this area, we introduced a relational spirituality model of personal sin that contextualizes a Christian’s post-transgression relational experience with God as part of a broader relational spirituality system whose elements may influence a person’s resolution of the transgression and experience of reconciliation with God. It is possible that this model may be applied to other theistic religious traditions as well since other theistic religions (e.g., Islam, Judaism) offer the possibility of a dynamic relationship with a potentially benevolent God (Silverman et al., 2016). Extending the relational spirituality model to nontheistic religions will take more work. One effort applied relational spirituality to Buddhism and Hinduism by affirming the importance of a real person in relationship with many divines (Lahood, 2010). While such an application does not encompass the notion of communion with one God that is inherent in the Christian tradition, it does recognize that relationality is part of different faiths and might be important to explore further in the context of divine forgiveness. In the sections that follow, we discuss the unique contributions of the proposed model to the psychological literature on divine forgiveness, its potential implications for research and practice with the Christian population, and its limits.

### Applying a systems lens captures relational spirituality dynamics

The relational spirituality model of personal sin is a dynamic systems approach that complements the linear framework proposed by Fincham and May (2023). While their components analysis outlines the sequential process of perceiving divine forgiveness, including conditional branching and feedback mechanisms, our model situates recognition of personal sin alongside key elements of a Christian’s relational spirituality system that are particularly pertinent to experiencing reconciliation with God in the aftermath of personal sin. In addition, a components analysis can only describe whether a component is present or absent; a dynamic systems approach can offer a potential framework for exploring what the implications of that absence might be.

Applying a systems approach to key proximal cognitive-affective appraisals within a person’s relational spirituality system can enhance our understanding of how Christians experience reconciliation with God following instances of personal sin. A systems perspective opens up a variety of potential avenues that have yet to be explored in the empirical literature on divine forgiveness, ranging from those that focus on specific aspects of the system (e.g., where in the system can dissonances be introduced?) to those that address how elements of the system are interrelated (e.g., how does the association between appraisals pertaining to the self and God change based on appraisals of the personal sin?) to those that consider the system more broadly (e.g., what pattern of associations within the relational spirituality system predicts a higher likelihood of experiencing reconciliation with God?).

The relational spirituality model of personal sin integrates a key element that has received limited attention in existing conceptual frameworks on divine forgiveness: the potential influence of other people. For example, whereas Fincham and May (2021, 2023) acknowledge the potential for other people (e.g., family members, religious community) to influence a person’s decision to seek divine forgiveness and perception of divine forgiveness, our proposed model allows a deeper exploration of these complex relationships. Appraisals pertaining to other people with direct or indirect ties to the personal sin may have complex linkages to different elements of the relational spirituality system. For instance, a Christian might experience absolution from God through a religious leader’s pronouncement of their absolution, which may change their perception of the conditions under which God’s forgiveness is available to other people. More positive perceptions of others, perhaps introduced with the support of a mental health professional, may help the person place more value on their relationships with others, leading them to have more positive feelings about themselves. Special attention to the role of other people in shaping a Christian’s experience of reconciliation with God opens up many potentially fruitful avenues for researchers to pursue, including which types of cognitive-affective appraisals about other people might be more likely to resolve, rather than maintain or exacerbate, the tension that was introduced into their relational spirituality system following recognition of a personal sin.

### Expanding the model to address more distal elements

The relational spirituality model of personal sin takes a focused approach by emphasizing the most salient proximal elements that are thought to shape experiences of reconciliation with God following personal sin. However, the model is flexible in that it allows for consideration of more distal factors that could impinge on a Christian’s relational spirituality system. One example is Christian theological differences about the practice of sacramental confession (i.e., a practice in which believers confess their personal sin to a priest and receive absolution). Consider the difference between Roman Catholicism and most Protestant denominations, such as the Methodist denomination. The former has a canonical expectation that members in good standing make a sacramental confession at least once a year, whereas the latter does not. Further, in certain Christian theologies, the priest represents God and the community in absolving an individual from personal sin and restoring their relationship to the Christian community, emphasizing the importance of addressing personal sin in relation to both God and the broader religious community. These norms might lead to differences in appraisals of elements of our proposed model. Because priests often function as a mediator between the self, the community, and God in Roman Catholicism, we might expect a stronger connection between the personal sin and other people nodes among those who identify as Roman Catholic compared to Methodists. Theological differences in norms may be important to consider in identifying factors that facilitate (or serve as barriers to) appraisals associated with adaptively resolving relational disharmony with God in the aftermath of personal sin.

A more distal example is cultural values. One dimension of culture that is frequently used to explore cultural differences in forgiveness is individualism vs. collectivism (Cook et al., 2021; Cowden, 2024; Sandage et al., 2020). Collectivistic cultures are more likely to be characterized by an interdependent self-construal that prioritizes the needs of the group and maintaining social harmony, whereas individualistic cultures are inclined to emphasize individuality and personal autonomy. These cultural differences might lead to cross-cultural variation in the pattern of relationships between the elements of the relational spirituality model of personal sin. For example, appraisals of the relationship between the personal sin and other people might be especially salient to the relational spirituality system of Christians in principally collectivistic cultures, whereas appraisals of the relationship between personal sin and the self may play a more prominent role in principally individualistic cultures. Our proposed model lays the foundation for empirical research to explore potential denominational or cultural differences in the structure of the network of interrelationships among the elements involved in shaping experiences of reconciliation with God among Christians.

### Appraisals allow for a complex experience to be measured

The relational spirituality model of personal sin focuses strictly on a Christian’s cognitive-affective appraisals of the self, the personal sin, God, and other people with a direct or indirect connection to the personal sin, as well as their appraisals of the relationships among each of these elements. While we have argued that appraisals involving these elements of the relational spirituality system are the central and most easily operationalized ingredients for understanding a Christian’s personal *experience* of reconciliation with God after a personal sin has introduced disharmony into their relationship with God, the relational spirituality dynamics involved in the experience of reconciliation with God are not reducible to the transgressor’s appraisals. For example, there may be people who alter the transgressor’s response to their personal sin without them being aware of this influence, or there may be specific amends-making behavior (e.g., offering an apology to those impacted negatively by the personal sin) that could emerge from appraisals. However, research into the interconnectedness of the elements in the divine forgiveness process requires a model that describes their interrelationships, and our proposed model takes an initial step toward addressing this need with respect to the Christian population. Cognitive-affective appraisals are a particularly useful way to think about these interrelationships since the appraisals take place internally. For instance, a Christian might understand what it means to be forgiven by God in common sense terms, but how do they come to experience a sense of absolute assurance that God has absolved them of the personal sin? Similarly, how should a Christian resolve the tension between absolution from God as a promise and amends-making as encouraged in scripture? These are questions that are worked out internally, even though there may be observable elements.

### Suboptimal spiritual resolution of disturbance to the system

When the relational spirituality system is disturbed by recognition of personal sin, prior work suggests that the dual processes of engaging in repentance with God and experiencing absolution from God are particularly adaptive (Cowden et al., in press). Both processes are considered necessary for improving the system’s homeostasis through the experience of reconciliation with God, which in turn opens the possibility for renewed or deeper communion with God. The story involving David and Nathan in 2 Samuel 12:1–13 offers one illustration of this. It is important to note, however, that experiencing reconciliation with God may not always be followed by a renewal or deepening of one’s sense of communion with God. For example, a Christian might be grappling with other forms of religious/spiritual struggles (e.g., existential concerns around ultimate meaning) that were present before becoming aware of personal sin and remain unresolved even after experiencing reconciliation with God. These ongoing struggles may interfere with the person’s sense of communion with God. Despite the continued struggles, experiencing reconciliation with God may remove key obstacles within their relational spirituality system, potentially creating positive ripple effects that support the renewal and strengthening of their communion with God.

There may be cases in which the processes of engaging in repentance with God, experiencing absolution from God, or both might be absent or unfulfilled (Cowden et al., in press). For example, a Christian may engage in repentance but not experience absolution from God (i.e., unexperienced absolution), experience absolution but not engage in repentance (i.e., unrepentant absolution), or neither process might be applicable (i.e., detachment). Although the relational spirituality dynamics involved in these cases will vary, in each case the disturbance that was introduced into the system is not likely to be resolved in a way that optimizes the potential for relational harmony with God. For example, a Christian might continue to experience high levels of guilt and shame despite engaging in repentance and being assured of God’s absolution. Others may experience generalized anxiety or unresolved grief that they do not realize is triggered by personal sin. More seriously, some may experience scrupulosity, a mental health condition characterized by extreme and unmanageable doubts and fears about religious issues (American Psychiatric Association, 2013). There may also be cases in which a person perseverates on elements of the personal sin or attempts to ignore it completely because it is too painful to encounter.

When such cases arise, mental health professionals and others who provide care to Christians (e.g., pastoral counselors)—within the constraints of their relationship with the individual and whether the person is in formal treatment or simply asking for advice—could play an important role in supporting adaptive resolution and improving stability in the relational spirituality system. For example, when working with someone who has engaged in repentance but is struggling to experience absolution from God, a spiritually competent therapist or pastor could listen nonjudgmentally to the wrongdoer, explore God’s forgiving character as described in the Bible, and remind them that moving from a state of unexperienced absolution can take time. They might also encourage the wrongdoer to explore spiritual disciplines that may be appropriate, such as reading scripture and prayer (Foster, 1998). There are passages in the Bible that deal explicitly with God’s forgiveness, particularly those that delineate the importance of repentance for personal sin (e.g., Mark 1:4, Luke 13:3). Practitioners could also consider employing relevant measures to assess the changes in key aspects of a person’s relational experience with God following recognition of personal sin, such as the Reconciliation with God Scale (Cowden et al., in press) and the Communion with God Scale (Knabb and Wang, 2021). In the liturgical Christian context (e.g., Anglicanism, Catholicism, Orthodoxy), the rites of confession, penance, and absolution can provide a tangible means for the communication of God’s mercy. For example, the act of kneeling before a priest and the rites of confession and absolution provide a visible, sacramental promise of forgiveness that can help ease a person’s internal feeling of remorse and encourage them to fully accept God’s forgiveness. Emphasizing the diverse sacramental styles that are available may help individuals find an approach that best meets their needs (Aihiokhai, 2020).

There may also be instances in which the instability in a Christian’s relational spirituality system is met with an attempt to bypass engaging in repentance or compensating for unexperienced absolution. For example, a person might blame others for the consequences of their personal sin as a defensive response that serves a self-protective function (e.g., preservation of self-image), but this avoidance response may become an obstacle to engaging in repentance. Although bypassing or compensating for these processes might not be optimally adaptive for experiencing communion and could negatively impact well-being over time, a Christian may experience temporary benefits that mask the underlying instability of the relational spirituality system. For instance, a person might respond to their personal sin by prioritizing relationships with people whose philosophies about morality contradict God’s values or standards, which may lead to the strengthening of some interpersonal bonds that could temporarily compensate for detrimental impacts of the personal sin on spiritual well-being. Our proposed model provides a framework for exploring some of the relational spirituality dynamics that may be involved in maintaining or exacerbating instability in the relational spirituality system, which could be useful for uncovering potential targets for intervention.

Even if the processes of engaging in repentance with God or the experience of absolution of God are not fulfilled at a particular point in time, it is worthwhile noting that there is often a temporal arc to both processes that may just need time to unfold (Cowden et al., in press). However, if the length of time either or both processes remain unfulfilled corresponds to lingering instability in the relational spirituality system, it is reasonable to expect that the negative implications of this state for a Christian’s sense of communion with God might trickle down to other dimensions of well-being (e.g., mental health).

### Broader implications of the model for well-being

In our proposed model, recognition of a personal sin destabilizes the relative homeostasis of the individual’s relational spirituality system. This type of religious/spiritual struggle may be accompanied by both “pain and gain” (Jung et al., 2022, p. 305). On the one hand, relational spirituality dynamics that lead to reconciliation with God will increase stability in the system that lays the foundation for renewed or strengthened communion with God (Cowden et al., in press). This represents a form of spiritual growth, which can be considered a ‘gain’ arising from the ‘pain’ of dealing with personal sin. Although we might situate these proximal benefits within the domain of spiritual well-being, more distal benefits to other domains of well-being might also arise because of the renewed communion with God that comes with increased stability in the relational spirituality system. For example, prior work has shown that communion with God is related to greater mental well-being (Knabb and Wang, 2021), and some evidence suggests that interventions for addressing religious/spiritual struggles may increase positive affect and decrease addictive behaviors (Reist Gibbel et al., 2019).

While we have been principally concerned with adaptive resolution of the disturbance to the relational spirituality system brought about by recognition of a personal sin, there are cases in which the instability introduced into the system goes unresolved and renewal of communion with God does not occur. Drawing on related empirical research on religious/spiritual struggles, such instances may have negative downstream implications for mental, social, and physical domains of well-being as well (Cowden et al., 2024a,b; Pomerleau et al., 2020). In cases when a Christian’s recognition of their personal sin is not accompanied by spiritual growth, our proposed model can point to some possible targets for intervention to support adaptive resolution of tension in the relational spirituality system and strengthen experiences of communion with God.

There is a rich literature addressing the idea that positive transformation and growth can occur as a consequence of adversity, crisis, and suffering (Tedeschi and Calhoun, 2004; Wong et al., 2022). This literature sheds light on the possibility that a brighter side (i.e., spiritual growth) can emerge following the discomfort of grappling with one’s personal sin. For example, the growth mindset literature identifies strategies by which one can ‘adopt’ a growth mindset (Ashford et al., 2020), suggesting that such a mindset can be acquired in the spiritual domain as well. Adoption of a growth mindset about religious/spiritual struggles might be especially beneficial to those who have engaged in repentance for a personal sin but are struggling to feel confident in God’s forgiveness. Strategies that a Christian might apply to grow spiritually from their personal sin might include viewing spiritual challenges as opportunities for personal growth (activating the ‘self’ node), practicing spiritual self-reflection through journaling about ways God has been good to them (activating the ‘God’ node), or deepening their membership in a religious community by attending weekly Bible study group meetings (activating the ‘other people’ node).

## Conclusion

The Christian spiritual journey inevitably involves confronting personal sin. Our proposed relational spirituality model of personal sin provides a conceptual framework for understanding how recognition of personal sin may disrupt the relative homeostasis of a Christian’s spiritual system and what key elements might help to improve stability within the system. While further research is needed to empirically validate our proposed model, Christians who desire deeper communion with God following recognition of personal sin may benefit from resources and supports that address different elements of their relational spirituality system. Promoting positive strategies for Christians to experience reconciliation with God following recognition of personal sin could strengthen their connection with God and enhance their well-being.

## Footnotes

^1^Although this kind of doctrinal belief is common among Christian denominations, there are some doctrinal differences as well. Our approach in this paper is to offer a broadly ecumenical conceptual model, though we do recognize the potential influence of denominational variation in beliefs and practices. At different points throughout this paper, we use examples to highlight some of these differences (e.g., liturgical vs. non-liturgical, Catholic vs. Protestant). Our intention is not to give an exhaustive list of possibilities but rather to give some representative examples for consideration, in part to avoid the possibility of obscuring convictions about God’s forgiveness that Christians hold in common.

^2^We use the term personal sin to signify that the subject is the individual whose thoughts, words, or actions have transgressed God’s standards or values (rather than, for example, a sin committed by another person that has repercussions for oneself). In this paper, our use of ‘personal’ is more theological—emphasizing that God has a direct, personal relationship with individuals (Grudem, 1994)—compared to its typical use in psychology, where ‘personal’ often implies individualism or self-focus (Rogers, 1961).

^3^In psychological terms, the term ‘self’ is a multifaceted dynamic construct that is shaped by both individual and social factors, including personal experiences, relationships, and societal norms (Baumeister, 2023). We use the term ‘self’ in the context of our proposed model to capture various self-perceptions, judgments, and inner responses that a transgressor may have following recognition of personal sin. Our use of the term ‘self’ is compatible with the relational perspective that characterizes Christian theology.

^4^The disciplines of theology and psychology both explore ways to promote human flourishing through their different disciplinary lenses. However, theology and psychology generally differ in how personhood (or self) is viewed. This paper is grounded in the discipline of psychology while appropriating some insights from theology. In Christian theology, human personhood is viewed as inherently relational (which stems from the idea that the Trinity—involving God the Father, Christ the Son, and the Holy Spirit—embodies relational personhood). Psychology is traditionally concerned with understanding human experience, such as how people think, feel, and behave. For various reasons (e.g., historical developments, methodological approaches), psychology is generally less firmly rooted in relationality than theology. Although our proposed relational spirituality model of personal sin is fundamentally psychological, it is an attempt to emphasize more strongly the relationality of personhood than is typically seen in psychology.

^5^It is noteworthy that the theological literature, in contrast to the psychological literature, frequently introduces the role of others in responding to personal sin. For example, the guidelines for communal living found in the writings of St. Benedict, St. Anselm, and the penitential books (or rulebooks for living the Christian life) emphasize communal responsibility in matters of repentance and absolution (Bieler, 1975; Leclerq, 1982). Some philosophical and theological perspectives also emphasize the relational nature of spirituality (Aihiokhai, 2025; Buber, 1937; Grenz, 2000).

^6^This passage is found in the Hebrew Bible. While it is interpreted similarly by both Jews and Christians, emphases differ. Our focus is on the Christian tradition, which generally emphasizes God’s grace and redemption. A Jewish interpretation might highlight David’s abuse of power, his subsequent need to repent in order to maintain covenant with God, and the importance of obedience to the law (Barton, 2022; Sanders, 1977).
